# The long non-coding RNA *PARTICLE* is associated with *WWOX* and the absence of FRA16D breakage in osteosarcoma patients

**DOI:** 10.18632/oncotarget.21086

**Published:** 2017-09-19

**Authors:** Valerie Bríd O'Leary, Doris Maugg, Jan Smida, Daniel Baumhoer, Michaela Nathrath, Saak Victor Ovsepian, Michael John Atkinson

**Affiliations:** ^1^ Institute of Radiation Biology, Helmholtz Zentrum Munich-German Research Center for Environmental Health, Neuherberg, Germany; ^2^ Department of Pediatrics and Children′s Cancer Research Center, Technical University Munich, Munich, Germany; ^3^ Bone Tumour Reference Center, Institute of Pathology, University Hospital Basel, Basel, Switzerland; ^4^ Pediatric Hematology and Oncology, Klinikum Kassel, Kassel, Germany; ^5^ Institute of Biological and Medical Imaging, Helmholtz Zentrum Munich-German Research Center for Environmental Health, Neuherberg, Germany; ^6^ Faculty for Electrical Engineering and Information Technology, Technical University Munich, Munich, Germany; ^7^ International Centre for Neurotherapeutics, Glasnevin, Dublin, Republic of Ireland; ^8^ Chair of Radiation Biology, Technical University Munich, Munich, Germany

**Keywords:** WW-domain, PARTICLE, osteosarcoma, FRA16D

## Abstract

Breakage of the fragile site FRA16D disrupts the *WWOX* (WW Domain Containing Oxidoreductase) tumor suppressor gene in osteosarcoma. However, the frequency of breakage is not sufficient to explain the rate of *WWOX* loss in pathogenesis. The involvement of non-coding RNA transcripts is proposed due to their accumulation at fragile sites, where they are advocated to influence specific chromosomal regions associated with malignancy. The long ncRNA *PARTICLE* (promoter of *MAT2A* antisense radiation-induced circulating long non-coding RNA) is transiently elevated in response to irradiation and influences epigenetic silencing modification within *WWOX*. It now emerges that elevated *PARTICLE* levels are significantly associated with FRA16D non-breakage in OS patients. Although not associated with overall survival, high *PARTICLE* levels were found to be significantly linked to metastasis free outcome. The transcription of both *PARTICLE* and *WWOX* are transiently responsive to exposure to low doses of radiation in osteosarcoma cell lines. Herein, a relationship between *WWOX* and *PARTICLE* transcription is suggested in human osteosarcoma cell lines representing alternative genetic backgrounds. *PARTICLE* over-expression ameliorated *WWOX* promoter activity in U2OS harboring FRA16D non-breakage. It can be concluded that the lncRNA *PARTICLE* influences the *WWOX* tumor suppressor and in the absence of *WWOX* FRA16D breakage, it is associated with OS metastasis-free survival.

## INTRODUCTION

Genetically unstable regions of the human genome frequently harbor tumor-suppressor genes [1, 2]. The second most frequently affected site of human chromosomal fragility is FRA16D located at 16q23 within the *WWOX* (tryptophan domain containing oxidoreductase) locus [3]. The sequences of both orthologous chromosomal fragile site regions for human FRA16D/*WWOX* and mouse Fra8E1/*Wwox* exhibit remarkable conservation [4, 5]. Deletion of *WWOX* in mice has resulted in the spontaneous development of osteosarcoma (OS) [6]. Likewise, studies have reported that the majority of human OS patients have undetectable WWOX protein levels [7, 8] or its compromised functionality in cases with metastasis [9]. Nevertheless, the lack of correlation between WWOX expression and osteosarcoma patient prognosis suggests that its loss is an early event in cancer pathogenesis with the phenotypic results of its deletion not contributing directly to patient demise [7]. The fact that *WWOX* deletion does not occur frequently but that loss of its expression is common suggests that factors influencing the epigenetic alterations of this gene might also be involved in the pathogenesis of osteosarcoma [7].

The involvement of non-coding RNA transcripts is proposed due to their reported accumulation at fragile sites, where they are believed to influence the structure of specific chromosomal regions associated with malignancy [10, 11]. Long non-coding RNA (lncRNA, transcripts longer than 200 nucleotides with limited coding potential) can induce epigenetic variations in the chromatin state of cancer cells [12]. Observed alterations in the abundance of many lncRNA transcripts in cancer, implicates their putative role in tumor pathogenesis [13]. Although seen as mainly regulators of gene expression, lncRNAs can also act via post-transcriptional mechanisms that are capable of influencing chromosome structure and stability [14].

The lncRNA *PARTICLE* (promoter of *MAT2A* antisense radiation-induced circulating long non-coding RNA [15]) is transcribed in the antisense direction within the promoter of methionine adenosyltransferase 2A (*MAT2A*), the product of which encodes the catalytic subunit of methionine adenosyltransferase [16]. *PARTICLE* forms a triplex within the *MAT2A* promoter and operates an active feedback silencing loop limiting the upregulation of *MAT2A* in response to low dose radiation [15]. In contrast to earlier models that showed *PARTICLEs* repressive ability was, typical of most lncRNAs, restricted to a specific gene at a local (usually in *cis*) level, it has been recently discovered that *PARTICLE* over-expression enhances trimethylation of histone H3 lysine 27 across the human genome with specific enrichment within *WWOX* [17, 18]. In contrast, diminished hyper-methylation of a *WWOX* promoter CpG island was evident upon *PARTICLE* knockdown [17]. Given the lack of FRA16D breakage and WWOX loss in some osteosarcomas we have investigated alternate mechanisms for suppressing *WWOX* expression. We hypothesized that the lncRNA *PARTICLE* might provide an alternative path for inactivation of *WWOX* in osteosarcoma where the fragile site remained intact.

## RESULTS

### *WWOX* and *PARTICLE* reveal contrasting expression patterns in osteosarcoma tumor samples

**Figure 1:**
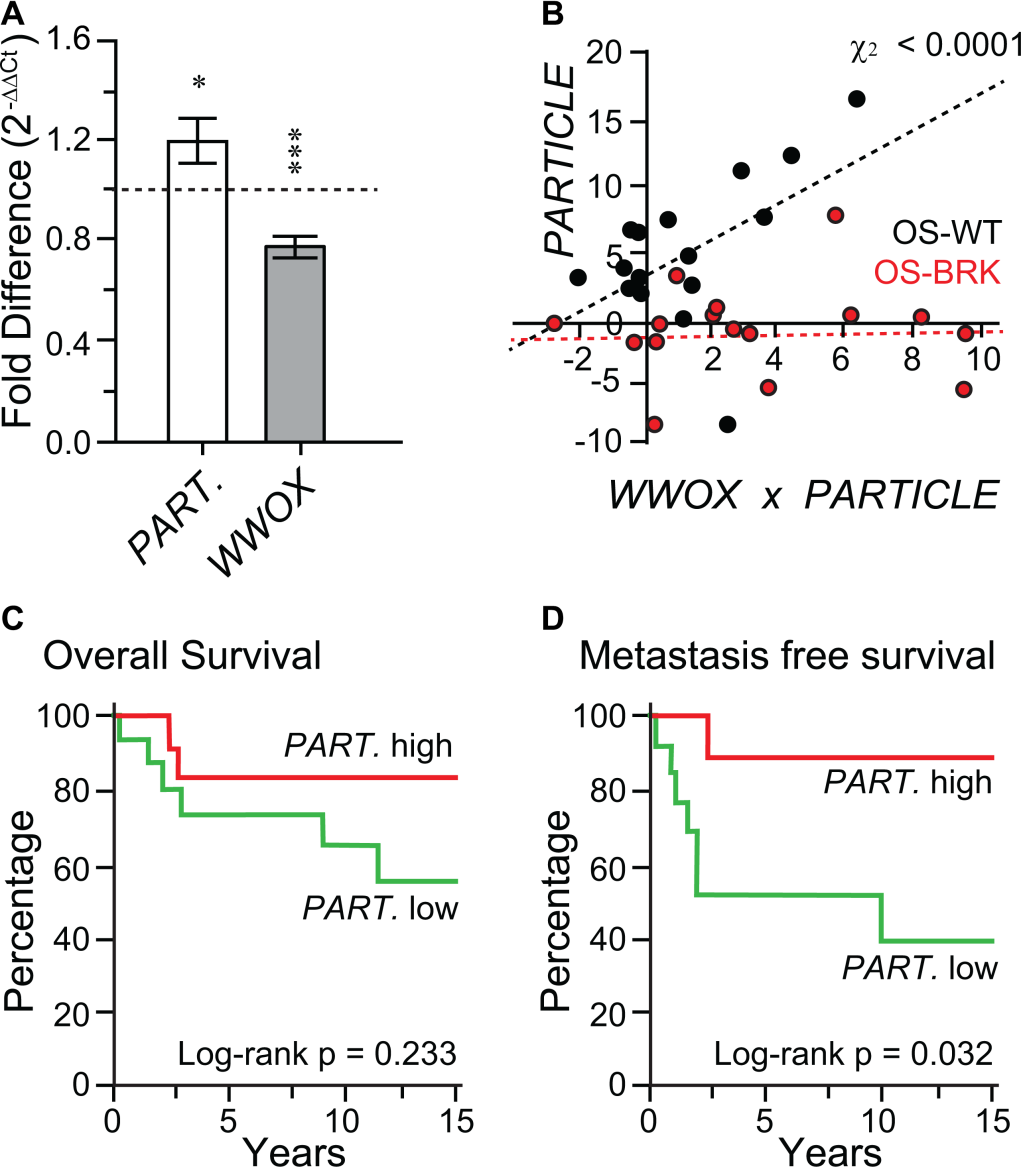
In the absence of FRA16D breakage the *WWOX* tumor suppressor is accompanied by elevated *PARTICLE* expression (**A**) Histograms depicting *PARTICLE* and *WWOX* expression in unselected osteosarcoma specimens relative to paired controls. (**B**) Scatterplot of Chi-square correlation between *PARTICLE* and *WWOX* in OS–WT (black dots) and OS–BRK (red dots). (**C** and **D**) Association of *PARTICLE* with overall survival (C) or metastasis free survival (D) amongst osteosarcoma patients. *PARTICLE* expression relative to the endogenous control gene encoding the TATA-binding protein in patient tumors was used for comparison with retrospective outcome. Event (defined as metastasis free survival) comparison using log-rank (Mantel-Cox) testing.

Differential *WWOX* and *PARTICLE* expression was determined in OS tumor samples and normal tissue controls [19] obtained from individuals of ~ 15 yr median age (*n* = 40, Table 1). *WWOX* was significantly down regulated (*p* = 0.000005) in OS tumor samples compared to controls. In contrast, *PARTICLE* expression was increased by 19.7 ± 0.8 % (*p* = 0.011) in OS tumor samples when similarly compared (Figure 1A). These findings revealed the distinct expression of *PARTICLE* in malignant tissue.

**Table 1:** Osteosarcoma clinical data from fourty patients partaking in this study

Osteosarcoma Patient Clinical Data	WWOX FRA16D Non-breakage (OS - WT)	WWOX FRA16D Breakage (OS - BRK)
**Gender**	***n* = 23**	*n* =17
Male	14	9
Female	9	8
**Age at Diagnosis (Years)**	***n* = 23**	***n* = 17**
Average	22.9	16.4
Median	15.3	14.9
Range	8 - 85	7 - 47
**Metastases**	*n* = 21	***n* = 15**
Yes	10	4
No	11	11
**Observation Period (Months)**	***n* = 22**	***n* = 17**
Average	4.9	6.4
Median	5.3	2.1
Range	0.3 – 12.2	0.2 - 17
**Response to Neo-adjuvants**	***n* = 21**	***n* = 15**
Good	13	7
Poor	8	8
**Survival**	***n* = 19**	***n* = 12**
Alive	15	8
Dead	4	4
Event^*^	***n* = 19**	***n* = 12**
Yes	8	4
No	11	8

### Absence of FRA16D breakage in the *WWOX* tumor suppressor is accompanied by elevated *PARTICLE* expression

Tumors were classified according to their FRA16D breakage status (non-breakage: OS-WT; breakage: OS-BRK). When these tumor groups were compared, highly significant increased *PARTICLE* levels were noted when the *WWOX* FRA16D was intact (Chi-square association *p* = 0.000059, Figure 1B). This positive correlation suggests that the absence of FRA16D breakage within the *WWOX* tumor suppressor is accompanied by elevated *PARTICLE* expression.

### *PARTICLE* expression is associated with metastasis free survival in osteosarcoma patients

No significant difference was found from Kaplan Mayer analysis of *PARTICLE* expression levels and overall patient survival (log-rank Mantel-Cox text *p* = 0.23, Figure 1C). Nevertheless, elevated *PARTICLE* expression was significantly associated with metastasis free survival in osteosarcoma patients within this sample cohort (log-rank Mantel-Cox test *p* = 0.0324; Figure 1D).

### Osteosarcoma cell lines elicit differential *PARTICLE* levels in response to radiation

**Figure 2:**
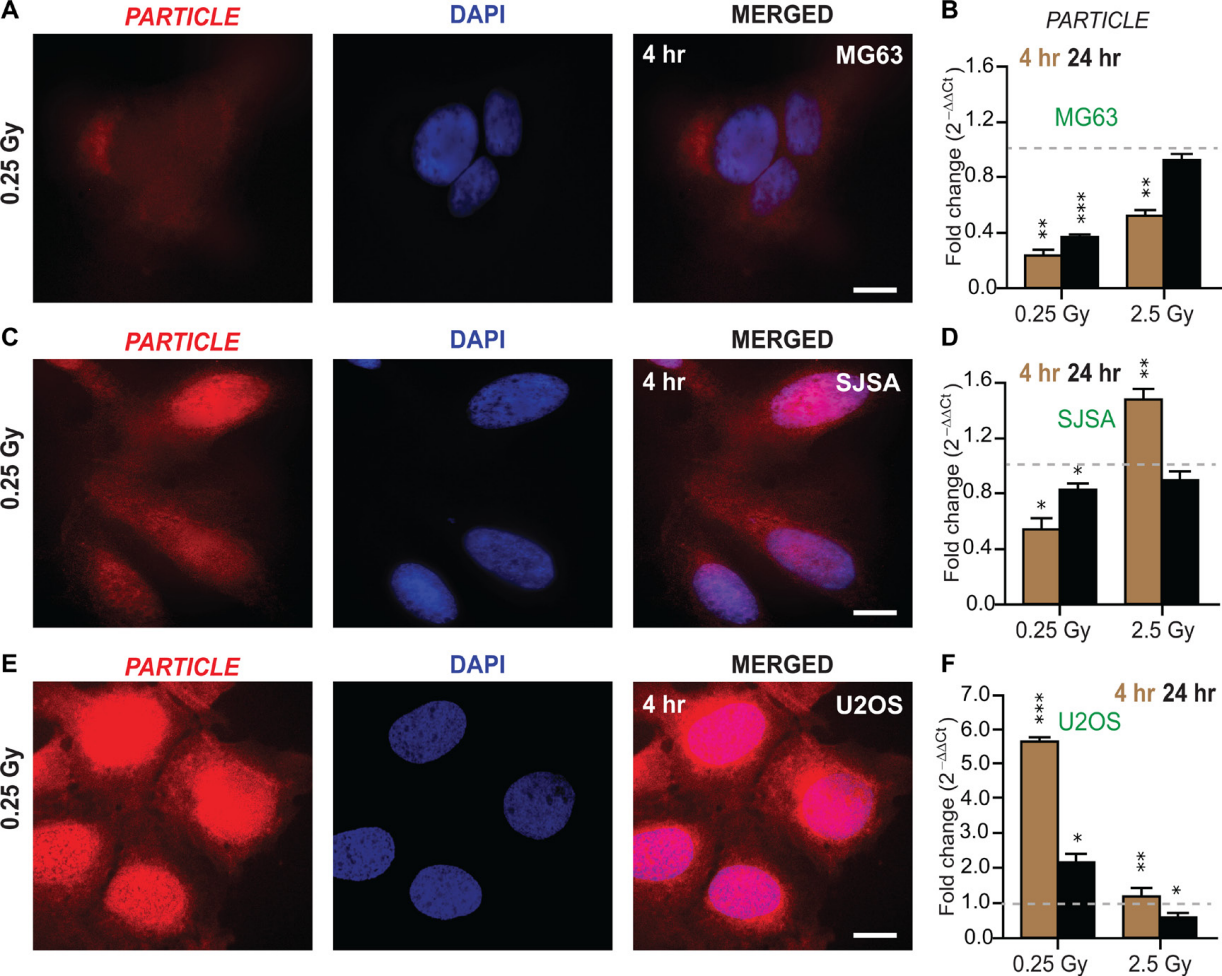
Differential *PARTICLE* expression in osteosarcoma cell lines in response to radiation (**A**, **C** and **E**) Representative epifluorescence microscopic images of MG63, SJSA and U2OS 4 hr post 0.25 Gy labelled with *in situ* hybridization probes (Quasar 570 (red)) specific for *PARTICLE* (left). Nuclei stained with DAPI (blue, middle). Merged images (right). Scale bar 10 μm. (**B**, **D** and **F**) Histograms of *PARTICLE* expression in osteosarcoma cell lines MG63 (B), SJSA (D) and U2OS (F) exposed 4 hr (brown bars) or 24 hr (black bars) post 0.25 or 2.5 Gy irradiation exposure.

*PARTICLE* transcript levels were examined in osteosarcoma cell lines (MG63, SJSA and U2OS, Figure 2) at 4 or 24 hr following low (0.25 Gy) or medium (2.5 Gy) dose radiation in comparison to sham-irradiated controls (Supplementary Figure 1). *In situ* hybridization revealed that *PARTICLE* transcripts were located pre-dominantly in the cytoplasm of MG63 post low dose irradiation (Figure 2A). Quantitative PCR analysis demonstrated a highly significant decrease in *PARTICLE* at 4 hr post low or medium irradiation (*p* = 0.002 and *p* = 0.005, respectively) with similar effects at 24 hr post 0.25 Gy (*p* = 0.005) (Figure 2B).

In SJSA, *PARTICLE* transcripts were more highly expressed in the nucleus in comparison to the cytoplasm (Figure 2C). In this cell line decreased *PARTICLE* expression was noted particularly at the 4 hr time point (*p* = 0.003) post 0.25 Gy (Figure 2D). In contrast, significant elevation in *PARTICLE* (*p* = 0.006, Figure 2D) was found 4 hr post exposure to medium dose irradiation (*p* < 0.0005, Figure 2D).

*In situ* hybridization, revealed that the intracellular distribution of *PARTICLE* in U2OS was predominantly nuclear 4 hr post 0.25 Gy exposure (Figure 2E). Ameliorated *PARTICLE* expression was noted (4.8 ± 0.6 fold increase) compared to sham irradiated cells 4 hr post low dose irradiation (*p* = 0.0002, Figure 2F). These findings demonstrate variable *PARTICLE* intracellular distribution and transcript levels post irradiation in representative osteosarcoma cell lines.

### *WWOX* transcript levels and promoter activity in osteosarcoma cell lines

**Figure 3:**
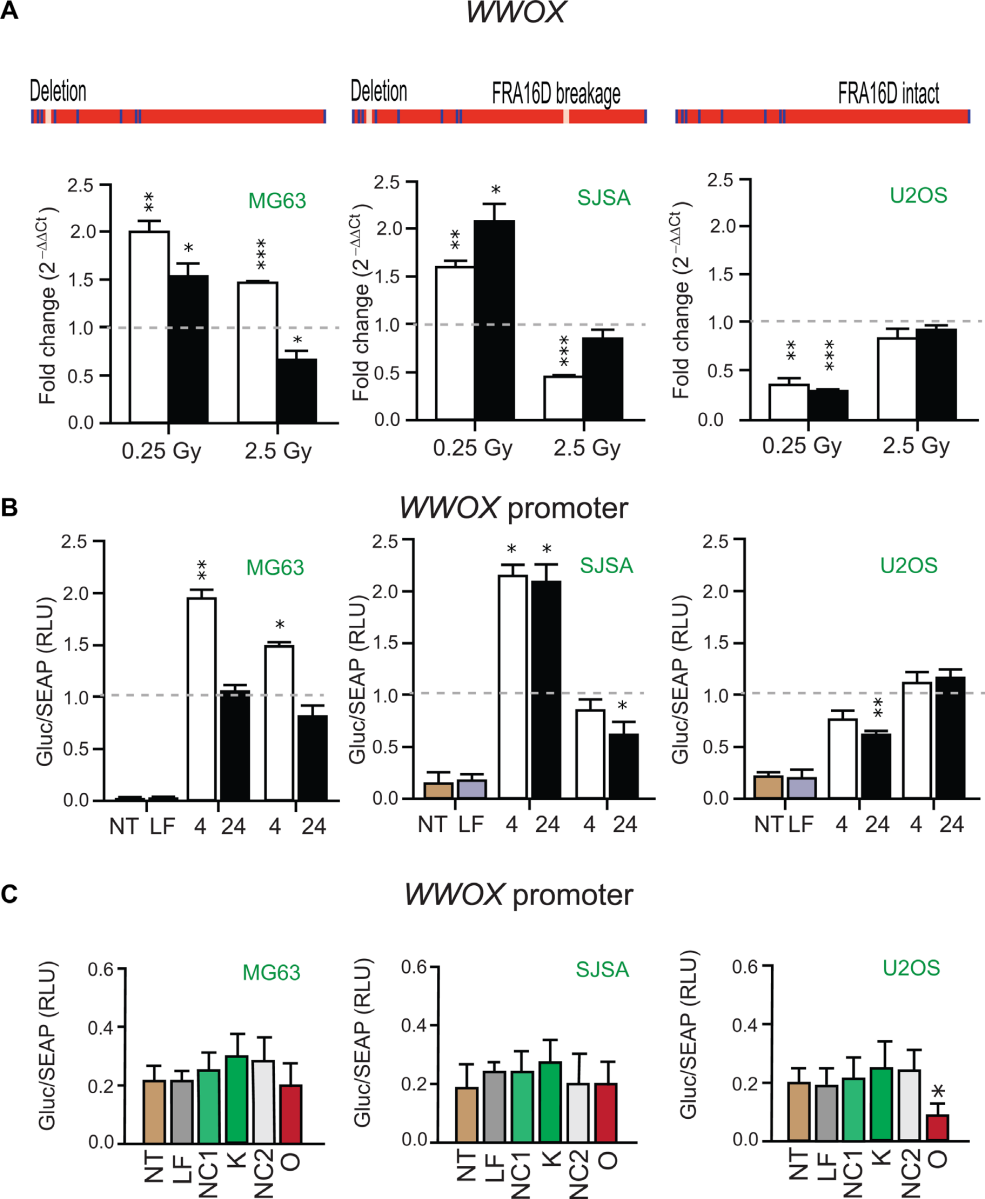
*WWOX* transcript levels and promoter activity in Osteosarcoma cell lines (**A**) Schematics of the *WWOX* gene in MG63, SJSA and U2OS with presence or absence of intron 3 deletion or FRA16D breakage indicated (above). Histograms of *WWOX* expression in these cell lines 4 hr (white bars) or 24 hr (black bars) post 0.25 or 2.5 Gy irradiation exposure. Values were normalized with TATA-binding protein (*TBP*) encoding the endogenous gene with relative expression comparison to sham-irradiated (0 Gy) equivalent cells (dashed lines). Data are represented as mean ± SEM (*n* = 3) with significance represented by asterisks (*p* < 0.05) where appropriate. (**B**) Histograms of *Gaussia* luciferase (GLuc) activity normalized to alkaline phosphatase activity (SEAP) indicative of *WWOX* promoter activity in MG63, SJSA and U2OS. Tissue culture media analyzed 4 hr and 24 hr following 0.25 Gy or 2.5 Gy cellular irradiation or sham irradiation (dashed line). Data are represented as mean ± SEM (*n* = 3). (**C**) Histograms of GLuc activity normalized to SEAP in non-transfected (NT), lipofectamine transfected (LF), *PARTICLE* knockdown (K), *PARTICLE* overexpression (O), siRNA negative control (NC1) or *in vitro* transcript control (NC2) in MG63, SJSA and U2OS. Data are represented as mean ± SEM (*n* = 3).

The osteosarcoma cell lines MG63, SJSA and U2OS are characterized by different *WWOX* genomic backgrounds [21]. MG63 and SJSA cells harbor a deletion in *WWOX* intron 3 (Figure 3A). In addition, *WWOX* contains FRA16D breakage in SJSA cells (Figure 3A). U2OS cells represent *WWOX* allelic imbalance and no FRA16D breakage in *WWOX* (Figure 3A). Following the revelation that *PARTICLE* was highly variable in these cell lines, comparison with *WWOX* expression and its promoter activity was instigated. These cell lines presented very different expression profiles for *PARTICLE* and *WWOX* at 4 or 24 hr following low (0.25 Gy) or medium (2.5 Gy) dose radiation exposure in comparison to sham-irradiated controls (Figures 2 and 3). In contrast to *PARTICLE* in MG63 (Figure 2B), significantly increased *WWOX* transcription was evident 4 hr post 0.25 or 2.5 Gy exposure (*p* = 0.0048 and *p* = 0.00005, respectively) in this cell line (Figure 3A). Luciferase reporter analysis of the *WWOX* promoter in MG63 also reflected significantly escalated activity at 4 hr post 0.25 or 2.5 Gy exposure after these dosages (*p* < 0.05) in comparison to sham irradiated controls (Figure 3B). In SJSA, a comparable pattern to MG63 emerged after low dose irradiation whereby increases in *WWOX* transcript levels (4 hr: *p* = 0.003; 24 hr: *p* = 0.01) and promoter activity (4 hr: p = 0.02; 24 hr: *p* = 0.05) were again accompanied by reduced *PARTICLE* levels (Figures 2B and 3A). Significant increases in *PARTICLE* (*p* = 0.006, Figure 2D) was likewise accompanied by reduction in *WWOX* expression 4 hr post exposure to medium dose irradiation (*p* < 0.0005, Figure 3A).

Diminished *WWOX* levels were detected in U2OS at 4 and 24 hr post low dose irradiation (*p* = 0.003 and *p* = 0.0004 respectively, Figure 3A). *WWOX* promoter activity was likewise reduced in this cell line particularly 24 hr post low dose irradiation exposure (*p* = 0.006, Figure 3B). Post irradiation the contrasting expression profiles of *PARTICLE* and *WWOX* in such cell lines that represented various *WWOX* genetic backgrounds suggest a relationship may exist between this lncRNA and this tumor suppressor gene.

### *PARTICLE* overexpression compromises *WWOX* promoter activity

Knockdown of *PARTICLE* expression did not significantly affect *WWOX* promoter activity in MG63, SJSA or U2OS (Figure 3C) in comparison to lipofectamine alone transfection or negative control (NC1). Overexpression of *PARTICLE* elicited different effects on *WWOX* promoter activity in these cell lines (Figure 3C). No significant decrease in *WWOX* promoter activity was noted in MG63 or SJSA (*p* > 0.05) when compared to lipofectamine alone transfection or an over-expressed ‘run-off’ transcript control (NC2). In U2OS, *PARTICLE* overexpression significantly reduced *WWOX* promoter activity by 55 ± 2 % (*p* = 0.013). Evidence is provided here that *WWOX* promoter activity is curtailed by the lncRNA *PARTICLE*.

## DISCUSSION

Herein, this report shows that higher *PARTICLE* expression levels are associated with malignant tissue and the absence of *WWOX* FRA16D breakage. Importantly, elevated levels of this lncRNA are significantly associated with metastasis free survival in osteosarcoma patients. Focus has shifted recently towards finding therapeutic biomarkers associated with metastasis-free survival as an alternative and important predictor of osteosarcoma outcome. Currently, patients with localized osteosarcoma have a 70% chance of 5-year prognosis. However, with the onset of metastasis or recurrent disease, patients have less than 20% chance of long-term survival despite aggressive therapies [22, 23]. A recent report [24] has shown that the provision of increased therapy to high risk patients was ineffective (ie. post 10 weeks operative and chemotherapy intervention), as was therapeutic intensity reduction for low risk patients. These prognostic outcomes for osteosarcoma have not changed significantly over the last 25 years.

It has been shown that osteosarcoma is the dominant phenotype in *WWOX* gene knockout mice [7]. Numerous studies have reported that *WWOX* alterations occur in many types of cancer cells and in situations of cellular stress [3]. Nonetheless, studies have failed to correlate the occurrence of genetic modifications in *WWOX* with cancer development, suggesting non-mutational regulation may occur [25]. Conflicting evidence exists related to the correlation between loss of WWOX protein and cancer prognosis [7, 26, 27]. Evidence has emerged that *PARTICLE* over-expression enhances the histone repressive modification mark across the human genome and specifically within *WWOX* in a breast cancer cell line [17]. *PARTICLE* was reported to increase the methylation status of a *WWOX* promoter CpG island (annotated CpG105476) of 990 bp located on chromosome 16: 78133076 – 78134066 (NCBI *homo sapiens* build number 37/hg19). The transcription initiation site for *WWOX* resides within this region at position chromosome 16: 78133327 orientated in a forward direction (NCBI gene id. 51741) [17]. It has also been demonstrated that *PARTICLE* overexpression leads to a reduction in *WWOX* expression [18].

Emerging evidence using bio-informatic tools from the latest miRbase and the ENCODE projects have revealed that chromosomal fragile sites are enriched in various non-coding binding elements, as well as specific variations in histone modifications [28]. This prompted us to investigate the association of elevated *PARTICLE* and an absence of FRA16D breakage in *WWOX* in tumor specimens. At the structural level, chromosomal fragile sites have the propensity to form secondary non-B DNA structures that interfere with the movement of the replication fork thus leading to its collapse and associated DNA breaks [29].

Intriguingly, transiently elevated expression of the lncRNA *PARTICLE* was found to accompany diminished *WWOX* transcript levels and promoter activity within *in vitro* models of osteosarcoma. Manipulation of *PARTICLE* transcript levels via siRNA interference or *in vitro* transcription and transfection demonstrated reduction of *WWOX* promoter activity in U2OS. Such an effect was absent from the other tested cell lines perhaps due to an alternative *PARTICLE* intracellular distribution in MG63 or the presence of FRA16D breakage in SJSA.

**Figure 4:**
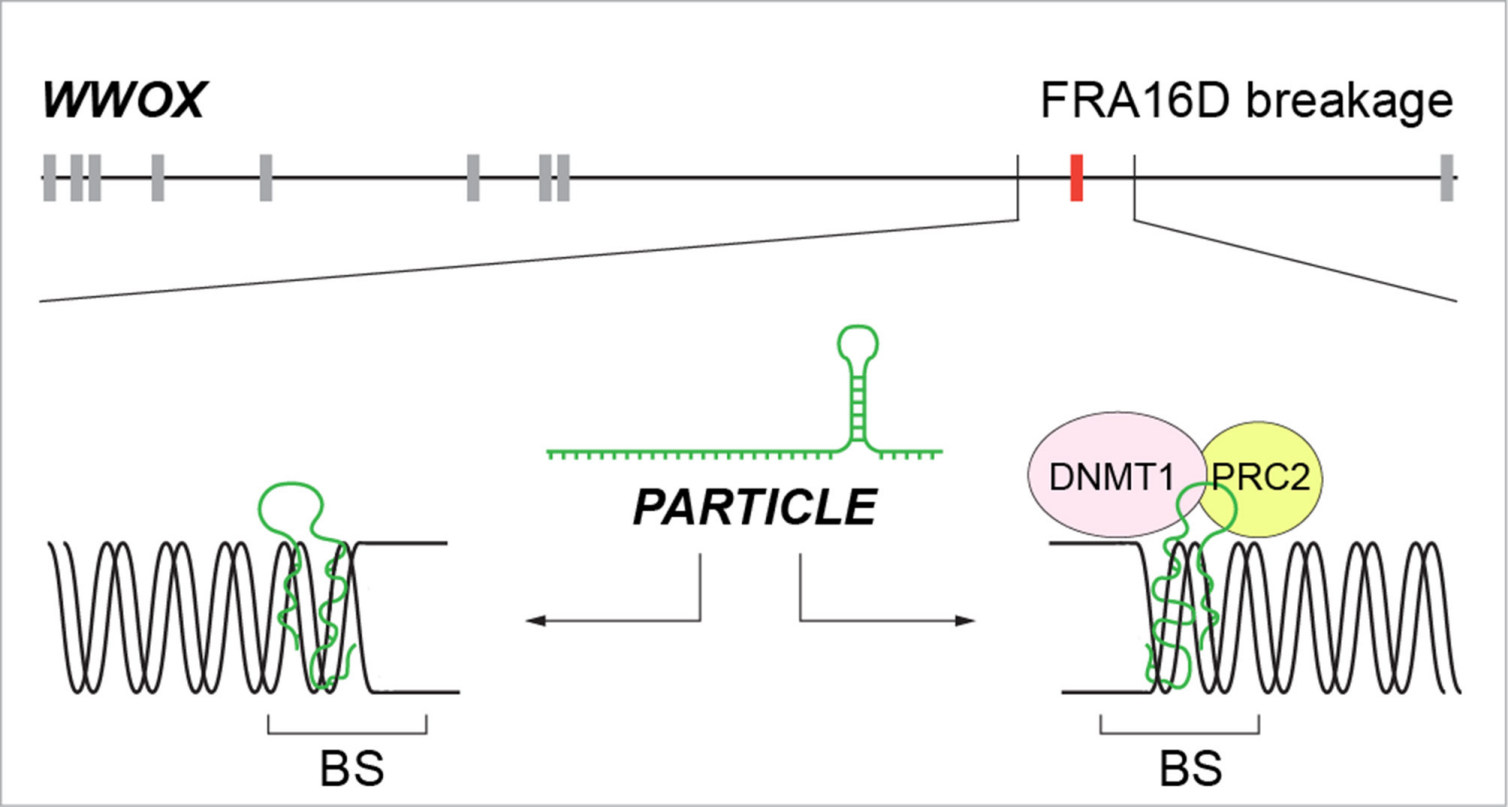
Schematic of the potential mechanisms by which *PARTICLE* association within the *WWOX* gene might avert FRA16D breakage yet hindering replication and transcription in tumor cells that lead to metastasis Replication fork remodeling (left) or scaffold formation (right) for epigenetic silencers. BS: breakage site; PRC2: Polycomb repressive complex 2; DNMT1: DNA methyltransferase 1.

It could be envisaged that *PARTICLE* association within the *WWOX* gene might avert FRA16D breakage through fork remodeling/scaffolding yet hindering replication and transcription in tumor cells that lead to metastasis (Figure 4). Rather than merely structural domains vulnerable to breakage, chromosomal fragile sites may be highly organized functional stress sensors cooperating with non-coding elements recruited to harness the effects of osteosarcoma progression.

## MATERIALS AND METHODS

### Ethical approval

This study was carried out in accordance with the recommendations of the medical faculty of the Technical University of Munich (TUM) with written informed consent from all subjects in accordance with the Declaration of Helsinki. The protocol was approved by the research ethics board (Reference 1867/07).

### Clinical information and osteosarcoma specimen preparation

Clinicopathological data (*n* = 40) were obtained from osteosarcoma patients with informed consent. Cases included 23 male and 17 female patients with osteosarcoma; mean age was 19.6 years. Metastases information was available for 90% of cases with 61% classified with local tumor status. The majority of patients (90%) had received neoadjuvant chemotherapy with 44% responding poorly to treatment. Anonymized primary tumors and paired controls [19] were obtained from this patient cohort (*n* = 40). All tumor samples were evaluated by an experienced bone pathologist who confirmed the diagnosis and the tumor content to be > 70% per sample. Total RNA was isolated from tumor tissue (~ 10 mg) or controls using an RNeasy mini-kit (cat # 74124, Qiagen).

### Propagation and maintenance of cell lines

U2OS, SJSA and MG63 human osteosarcoma cell lines (American Type Culture Collection) were cultivated in Roswell Park Memorial Institute (RPMI) 1640 media (GibcoTM cat # 21875-034) and fetal bovine serum (10%) at 37°C in a humidified incubator containing 95% oxygen/5% carbon dioxide atmosphere. The identity of these cell lines was verified by microsatellite analysis (Forensik GmbH, Germany). All cultures were routinely checked for mycoplasma contamination using a MycoAlert mycoplasma detection kit (Lonza, cat. # LT07-218). In general, cells were grown to 80% confluency prior to removal from the dish using trypsin (0.25%)/ EDTA (0.02%) and sub-culturing or harvesting.

### RNA interference targeting *PARTICLE*

*PARTICLE* knockdown was undertaken with Silencer^®^ Select siRNA interference technology (siRNA id: n307629; Part # 4390771). U2OS, SJSA and MG63 were grown to 60% confluence and transfected with these siRNAs (10 nM) using lipofectamine as per manufacturer instructions. After 72 hr, cells were transferred onto 6 well dishes for *WWOX* promoter luminescence assay determination (see below). Control conditions included transfection with lipofectamine and/or negative siRNA (NC1; cat # AM4615 no.3, Thermo Scientific).

### *PARTICLE* overexpression

*PARTICLE* was cloned into the pGEM^®^ - T vector (p.*PART*) (GenScript) and transformed into Top10 bacteria. A colony was grown in ampicillin (100 mg/ml) overnight and plasmid midiprep (Promega) performed. Plasmid concentration and purity was assessed (NanoDrop 1000, Thermo Scientific) with A260/280 ratio determination with automated sequence validation (GenScript). Plasmid linearization was carried out using 1mg plasmid DNA and S*ac*I overnight digestion at 37°C. *PARTICLE* (1432bp) was *in vitro* transcribed from a pGEM^®^-T vector (GenScript) using the TranscriptAid T7 High Yield transcription kit (Thermo Scientific, cat # K0441). Transcripts were treated with RNase-free DNase 1 (Thermo Scientific) and purified using an RNeasy mini-elute cleanup kit (Qiagen, cat # 74204) and verified by TBE-agarose (1.8%) electrophoresis. Prior (24 hr) to transfection, U2OS, SJSA and MG63 were seeded (10^5^ cells/well in a 6 well dish) in growth media (described above) in the absence of antibiotic/anti-mycotic to ~ 70% confluence at the time of transfection. The control template included in the Transcript T7 High Yield Transcription kit (Thermo Scientific, cat # K0441) as utilised for the production of a 2223bp ‘run off’ transcript serving as a negative control (NC2) for over-expression studies. Cells were transfected with lipofectamine and *PARTICLE* (4 mg) or negative control (4 mg) as per standard conditions with incubation for 72 hr prior to *WWOX* promoter luminescence assay.

### Irradiation

All irradiation was performed using a closed HWM-D 2000 Cesium^137^ source (Wälischmiller Engineering GmbH, Markdorf, DE; 10 cm height, 33 cm diameter) at a dose rate of 0.0082Gy/sec. Sham irradiation of controls involved only transport to the irradiation facility.

### RNA isolation and cDNA synthesis

Total RNA was isolated from cell lines and purified using TriFast peqGOLD (Peqlab, cat # 30–2010) and a Maxwell^®^ 16 LEV Blood DNA kit (Promega cat # AS1290) with solution substitution (ie. isopropanol replacement by 100 % ethanol in cartridge number 1) and a Maxwell^®^ 16 machine (Promega). The final elution was in ultra-pure water with concentration and purity assessment using O.D. 260/280 ratio determination (NanoDrop 1000, Thermo Scientific). Total RNA was stored at −80°C. Total RNA (1 mg) from sham irradiated or irradiated cells was converted into first strand cDNA using standard protocol procedures (with the inclusion of random hexamers and oligo dT primers) and reagents from Life Technologies, Germany.

### Real time PCR quantification

*PARTICLE* and *TBP* primers and probes [15] or pre-designed *WWOX* (Hs00249590_ml: single Taqman gene expression assay spanning the exon 1–2 boundary) (ThermoFisher cat # 4331182) were utilized for gene expression determinations. The reaction conditions for single gene assays were as such: cDNA (50–100 ng), 1× Taqman universal PCR master mix (no AmpErase UNG; Life Technologies, cat. # 4324018), forward and reverse primers (10 pmol), specific fluorescent probe (5 pmol), nuclease-free water up to 25 ml. For pre-designed assays, similar conditions were used except for utilization of an assay mix (1 X) instead of individual primers and probes. When pre-amplification was necessary (following total RNA extraction from human osteosarcoma and control specimens), a pooled assay mix was initially prepared (1 pmol primers and 0.5 pmol probes) for *PARTICLE*, *WWOX* and *TBP* (endogenous control) genes. Pre-amplification conditions were prepared as per manufacturer instructions (Life Technologies, cat # 4384556) and performed as follows: 95°C 10 min (1 cycle), 95°C 15 sec and 60°C 4 min (14 cycles), 4°C hold. Cycle threshold values were extracted and fold changes in gene expression determined by 2^(−ΔΔCt)^ [20]. Negative controls were normalized to a value = 1 and samples relatively compared.

### *WWOX* promoter luminescence assay

MG63, SJSA and U20S were plated on 6 well dishes (2 × 10^5^ cells/well) in growth media (2 ml) (above). After 24 hr cells were transfected as per manufacturer's instructions with a *WWOX* GLuc-on promoter reporter clone (GeneCopoeia, Rockville, MD, USA, cat. # HPRM19966-PG04) cloned into pEZX-LvPG04. This vector contains reporter and tracking genes encoding *Gaussia* luciferase (GLuc) and alkaline phosphatase (SEAP) secreted reporter proteins, respectively. Cells were irradiated 72 hr after transfection using lipofectamine. Culture medium (100 ml) was collected for analysis 4 hr and 24 hr after 0.25 Gy or 2.5 Gy irradiation exposures. Cell culture medium was also collected from sham-irradiated +/− lipofectamine transfection as controls. In addition, a positive control (EF1A-PG04, GeneCopoeia, Rockville, MD, USA) was assessed in parallel for verification purposes. The GLuc assay was conducted using buffer GL-S (GeneCopoeia, Rockville, MD, USA) for stable activity. Duplicate luminescence measurements were obtained with 3 second integration using white opaque 96 well flat bottom plates (Becton Dickinson Labware Europe, cat. # 353296). Signal normalization was done using the SEAP signal as an internal standard control (ratio of GLuc/SEAP activities) to eliminate the impact of variation in transfection efficiency.

### *PARTICLE in situ* hybridization and microscopic analysis

Procedures were followed as previously reported [15] and in accordance with Stellaris fluorescence *in situ* hybridization (FISH) (Biosearch Technologies) website information (www.biocat.com). Using the online probe designer tool (www.biosearchtech.com/stellarisdesigner/), 40 specific probes were selected from an input sequence (*PARTICLE* NR_038942.1) for optimal binding properties to the target RNA sequence. Search parameters were selected that included a masking level (3–5), maximum probe coverage = 40 and minimum 2 nucleotides spacing level. The probe fluorophore Quasar 570 (Ex: 552 nm; Em: 570 nm) was used for *PARTICLE* detection. Cells were mounted in VECTASHIELD™ HardSet™ containing the nuclear counterstain DAPI (Vector lab, cat # H-1500). *PARTICLE* or nuclear images were acquired using a TexasRed or DAPI (Ex/Em: 350/470 nm) dual filter wheel respectively on an inverted Axiovert 200 (Zeiss) fluorescence microscope with apotome slide module activation.

### Statistical analysis

Values are expressed as the mean ± S.E.M., with *n* = 3 referring to the number of independent biological replicates. Comparison of means or groups were tested using the Student's *t-test* or ANOVA with *p* values < 0.05 taken to indicate statistical significance. Comparisons of frequencies were performed using the Chi square test using GraphPad Prism statistical analysis software. Clinical parameters included metastasis or osteosarcoma cause-specific mortality. The time to occurrence of an event was computed from the date of diagnosis to the date when the event was first recorded. Kaplan-Meier survival analysis was used to examine the relationship between overall survival or metastasis-free survival and *PARTICLE* expression. High or low expression levels of *PARTICLE* were considered either above or below a value of 1 respectively following normalization. A log-rank Mantel-Cox test was used (GraphPad Prism) to test the statistical significance of the differences in survival rate estimators.

## SUPPLEMENTARY FIGURE


