# Supplementary material for: The long non-coding RNA PARTICLE is associated with WWOX and the absence of FRA16D breakage in osteosarcoma patients
Source: Oncotarget. 2017 Sep 19;8(50):87431–41. doi: 10.18632/oncotarget.21086 (PMC5675644; doi:10.18632/oncotarget.21086)
Supplement: Supplementary file 1 [file oncotarget-08-87431-s001.pdf]

The long non-coding RNA *PARTICLE* is associated with *WWOX* and the absence of FRA16D breakage in osteosarcoma patients

SUPPLEMENTARY MATERIALS

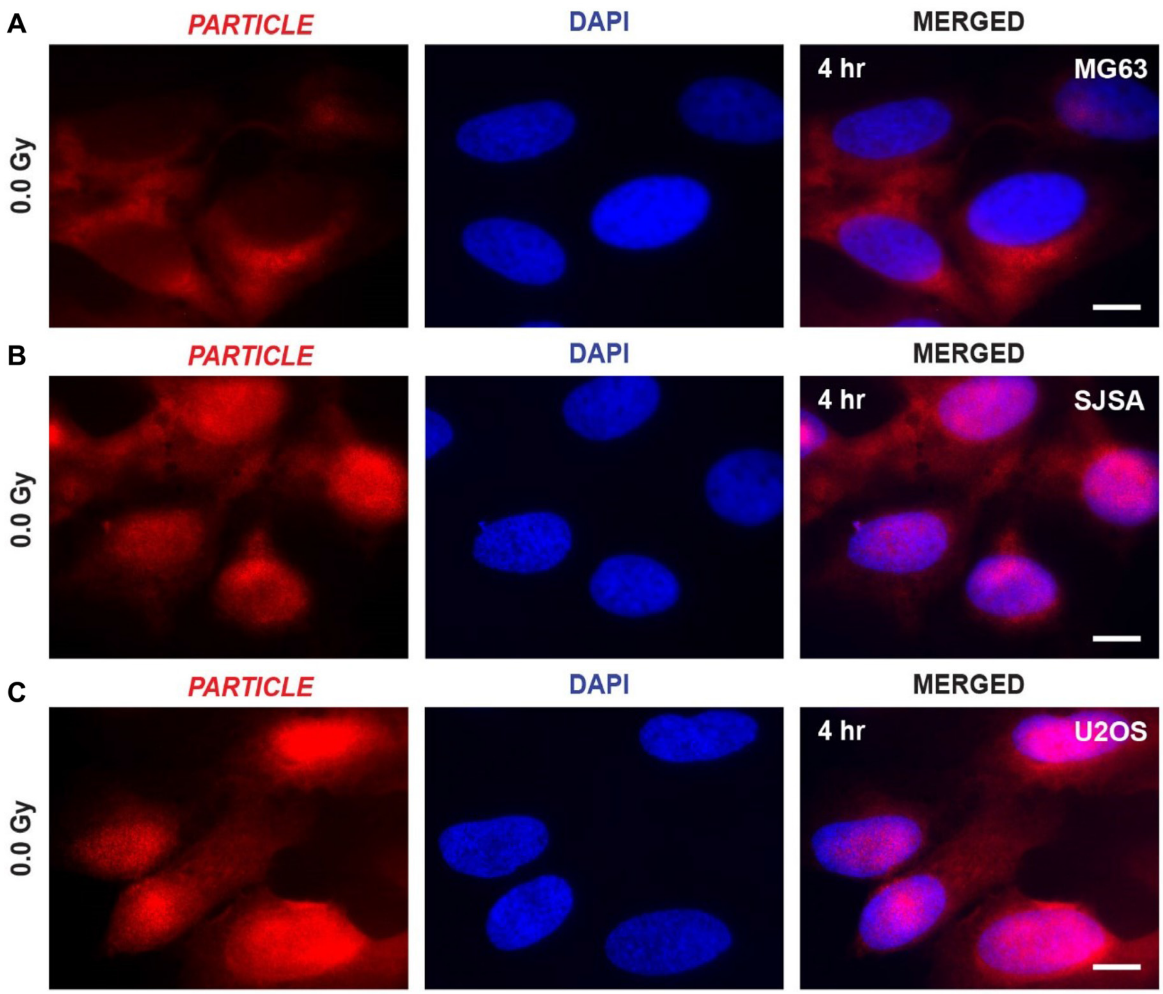

Supplementary Figure 1: *PARTICLE* expression in osteosarcoma cell lines. (Related to Figure 2) Representative epifluorescence microscopic images of MG63 (**A**), SJSA (**B**), and U2OS (**C**) labelled with *in situ* hybridization probes (Quasar 570 (red)) specific for *PARTICLE* (left). Nuclei stained with DAPI (blue, middle). Merged images (right). Scale bar 10 µm.
